# Viruses of free-roaming and hunting dogs in Uganda show elevated prevalence, richness and abundance across a gradient of contact with wildlife

**DOI:** 10.1099/jgv.0.002011

**Published:** 2024-07-24

**Authors:** Dickson S. Tayebwa, David Hyeroba, Christopher D. Dunn, Emily Dunay, Jordan C. Richard, Savino Biryomumaisho, James O. Acai, Tony L. Goldberg

**Affiliations:** 1 Department of Veterinary Pharmacy Clinical and Comparative Medicine, School of Veterinary Medicine and Animal Resources, Makerere University, P.O. Box 7062, Kampala, Uganda; 2 Department of Pathobiological Sciences, School of Veterinary Medicine, University of Wisconsin-Madison, 1656 Linden Drive, Madison, Wisconsin, 53706, USA

**Keywords:** Africa, ecology, epidemiology, free-roaming dogs, Uganda, viruses, zoonoses

## Abstract

Domestic dogs (*Canis lupus familiaris*) live with humans, frequently contact other animals and may serve as intermediary hosts for the transmission of viruses. Free-roaming dogs, which account for over 70% of the world’s domestic dog population, may pose a particularly high risk in this regard. We conducted an epidemiological study of dog viromes in three locations in Uganda, representing low, medium and high rates of contact with wildlife, ranging from dogs owned specifically for traditional hunting in a biodiversity and disease ‘hotspot’ to pets in an affluent suburb. We quantified rates of contact between dogs and wildlife through owner interviews and conducted canine veterinary health assessments. We then applied broad-spectrum viral metagenomics to blood plasma samples, from which we identified 46 viruses, 44 of which were previously undescribed, in three viral families, *Sedoreoviridae*, *Parvoviridae* and *Anelloviridae*. All 46 viruses (100 %) occurred in the high-contact population of dogs compared to 63 % and 39 % in the medium- and low-contact populations, respectively. Viral prevalence ranged from 2.1 % to 92.0 % among viruses and was highest, on average, in the high-contact population (22.3 %), followed by the medium-contact (12.3 %) and low-contact (4.8 %) populations. Viral richness (number of viruses per dog) ranged from 0 to 27 and was markedly higher, on average, in the high-contact population (10.2) than in the medium-contact (5.7) or low-contact (2.3) populations. Viral richness was strongly positively correlated with the number of times per year that a dog was fed wildlife and negatively correlated with the body condition score, body temperature and packed cell volume. Viral abundance (cumulative normalized metagenomic read density) varied 124-fold among dogs and was, on average, 4.1-fold higher and 2.4-fold higher in the high-contact population of dogs than in the low-contact or medium-contact populations, respectively. Viral abundance was also strongly positively correlated with the number of times per year that a dog was fed wildlife, negatively correlated with packed cell volume and positively correlated with white blood cell count. These trends were driven by nine viruses in the family *Anelloviridae*, genus *Thetatorquevirus*, and by one novel virus in the family *Sedoreoviridae*, genus *Orbivirus*. The genus *Orbivirus* contains zoonotic viruses and viruses that dogs can acquire through ingestion of infected meat. Overall, our findings show that viral prevalence, richness and abundance increased across a gradient of contact between dogs and wildlife and that the health status of the dog modified viral infection. Other ecological, geographic and social factors may also have contributed to these trends. Our finding of a novel orbivirus in dogs with high wildlife contact supports the idea that free-roaming dogs may serve as intermediary hosts for viruses of medical importance to humans and other animals.

**Impact Statement**

Free-roaming dogs are ubiquitous worldwide and have frequent, direct contact with people and other animals. We examined dogs in three populations in Uganda, spanning a gradient of wildlife contact, from dogs owned specifically for traditional hunting to pampered pets in an affluent suburb. Applying broad-spectrum DNA sequencing to blood samples, we identified 46 viruses, 44 of which were previously unknown. Viral infection increased as the rate of wildlife contact increased across populations. One virus, a novel orbivirus, has relatives that dogs can acquire by eating infected meat, and other relatives of this virus are zoonotic. Our study confirms that free-roaming dogs are at an elevated risk of viral infection in areas of high wildlife contact, including with viruses that merit attention as possible zoonoses. Monitoring free-roaming dogs and their owners in high-risk areas might be an efficient early warning system for detecting spillover.

## Data Availability

All raw sequence reads were deposited in the NIH National Center for Biotechnology Information (NCBI) Sequence Read Achieve under BioProject PRJNA1060647 (accession numbers SRR27429231-SRR27429273). All assembled virus genome sequences were deposited in NCBI GenBank under accession numbers PP105463-PP105508.

## Introduction

Of the hundreds of millions of dogs in the world, over 70 % are free-roaming [1]. Free-roaming dogs live fully or partially in human habitats in self-reproducing populations but are not permanently under human control and may or may not have owners [2]. In addition to causing problems related to animal welfare and population control [1], these dogs pose multiple risks to humans, livestock and wildlife. Such risks include bites [3,4], depredation of livestock [5], hybridization with wild canids [6,7], competition with wildlife [6], road accidents [8] and disease transmission [9,12].

Free-roaming dogs host an ever-expanding litany of zoonoses, many of which are viral [12,15]. Chief among these is rabies, with dogs annually responsible for up to 99 % of human cases globally, 59 000 human deaths, over 3.7 million disability-adjusted life years and 8.6 billion USD economic losses (estimates from 2015) [16]. Free-roaming and pet dogs have also been implicated in the transmission of several globally important emerging viral zoonoses. For example, dogs can occasionally be infected with SARS-CoV-2, leading to concerns about risks of onward transmission, but such infections appear to be mild and are likely not transmitted further [17,19]. Initial concerns about dogs contracting ebolaviruses and transmitting them to humans appear to be unfounded, despite evidence of seroconversion of dogs in heavily affected locations [20,21]. Less controversial is the strong evidence that free-roaming dogs can transmit viral diseases such as rabies and distemper to wild carnivores, in some cases severely hampering conservation efforts [22,24].

Because free-roaming dogs live with people and frequently contact wildlife, they may function as intermediary hosts for zoonoses [12,25,27]. Empirical support for this idea is scant in the case of viruses, however. To fill this knowledge gap, we undertook an epidemiological and virological investigation of free-roaming dogs across a gradient of wildlife contact in Uganda. We sought to establish whether viral infection correlates with the extent of dogs’ interaction with wildlife and, if so, whether viruses driving such a trend were likely to have been acquired from wildlife. We studied dogs in three locations in which they had low, medium and high rates of contact with wildlife. We gathered health data on dogs, quantified wildlife contact rates and applied broad-spectrum viral metagenomics to blood plasma samples. Our findings affirm that interaction rates between dogs and wildlife predict the diversity and types of viruses present in dogs and that dogs with high rates of wildlife contact host novel and potentially zoonotic viruses.

## Methods

### Study locations

Uganda has a large population of free-roaming dogs and a high prevalence of canine rabies [26,28]. Uganda also has diverse and abundant wildlife, much of which exists within its national parks, game reserves and forest reserves [29]. Some of Uganda’s wildlife-rich areas are also ‘hotspots’ for viral emergence, for example, Marburg virus and several ebolaviruses [30,31]; Crimean Congo haemorrhagic fever virus [32]; a variety of arboviruses, many of which were originally discovered in Uganda [33,38], and many lesser-known and novel viruses with high predicted zoonotic potential [39,43].

**Fig. 1.**
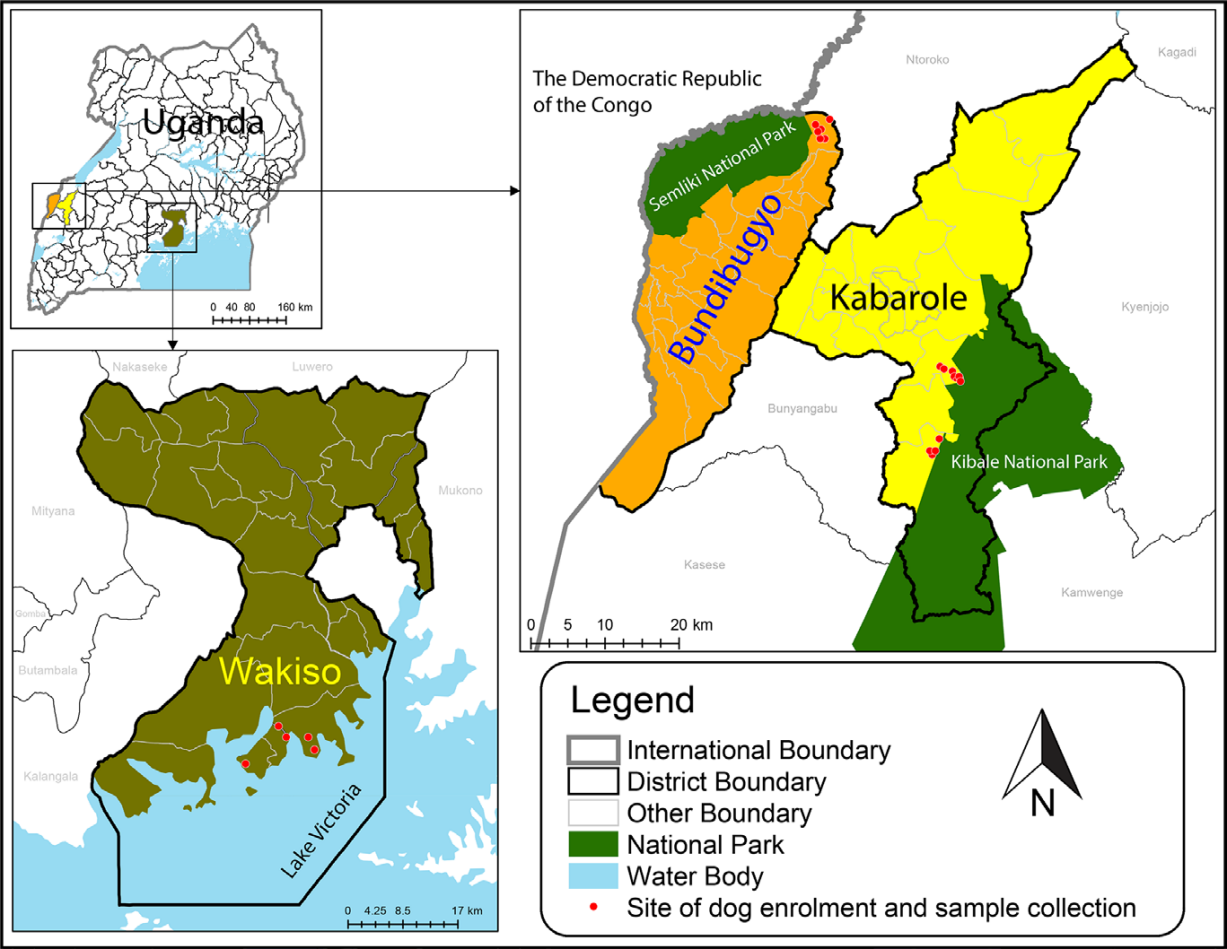
Map of study locations, showing three districts in Uganda chosen to span a gradient of contact between dogs and wildlife (Bundibugyo District, high contact; Kabarole District, medium contact and Wakiso District, low contact).

We conducted the study in three locations in Uganda selected to represent a gradient of contact between dogs and wildlife (Fig. 1). Bundibugyo District, the location of the high-contact study population, is in the far western part of Uganda, bordering the Democratic Republic of the Congo. Dogs selected for this study were from villages adjacent to Rwenzori National Park, on the border between Uganda and the Democratic Republic of the Congo [44], and were specifically owned for traditional wildlife hunting by members of the Bwamba and Bakonjo ethnic groups [45]. Encounters between dogs and wildlife in this context are extremely violent, involving regular exposure to blood and, in many cases, consumption of wildlife by dogs [26]. This area is also a hotspot for ebolavirus disease outbreaks, with one virus, Bundibugyo ebolavirus, named after this district following its first detection there in 2007 [46]. Bundibugyo District was also the location of a 2022 outbreak of Sudan ebolavirus [30], and cases of Zaïre ebolavirus were detected in the bordering Kasese District in 2019 [47].

Kabarole District, the location of the medium-contact study population, borders Bundibugyo District but is on the eastern side of the Rwenzori Mountains, making it part of a separate biogeographic region. It is inhabited principally by the Batooro, Batuku, Basongora and Bakiga ethnic groups, whose main economic activities are subsistence agriculture and animal husbandry [45]. Part of Kabarole District is covered by Kibale National Park, which is a semideciduous montane forest known for its diversity and biomass of nonhuman primates and other wildlife [48]. Dogs selected for this study in Kabarole District were not used for traditional hunting but were owned as guard dogs and pets, and they did have occasional (and usually violent) interactions with wildlife at forest edges and in agricultural fields [26,49].

Wakiso District, the location of the low-contact study population, encircles Kampala, Uganda’s capitol, is the most populous district in Uganda and is a highly urbanized environment. For this study, we enrolled dogs in Entebbe, an affluent suburban town near Kampala. The dogs were owned as pets and guard dogs and were selected from among dogs presented for routine preventive care to a local small animal veterinary clinic. These dogs had very limited or no access to wildlife, were generally not free-roaming and served as a comparison group.

### Enrolment, health evaluation and blood collection

We selected households at random from among those enrolled in a long-term study of human and animal health [25]. Within households that agreed to participate, we enrolled all adult and subadult dogs.

After obtaining client consent, we completed an owner survey for every dog enrolled. The survey gathered information on age, health history, vaccination and anthelminthic prophylaxis. We also collected data on whether the dog hunted wildlife and the number of times per year that a dog was fed wildlife. At the time of enrolment, we conducted a physical examination of each dog, which included the body condition score (BCS) estimated on a scale of 1 (very thin) to 5 (obese) [50] and body temperature measured using a rectal thermometer. We then obtained a sample of venous blood (1–2 ml) from the cephalic vein into evacuated tubes containing EDTA for clinical pathology (Vacutainer Plus Tubes, Becton, Dickinson and Company, Franklin Lakes, NJ, USA) and into plasma separation tubes for molecular assays [plasma preparation tubes (PPTs), Becton, Dickinson and Company]. We used blood preserved in EDTA to measure packed cell volume (PCV; per cent) and total protein (TP; grams per decilitre) on site and to make thin smears onto microscope slides for white blood cell counts (WBCs; cells per microlitre), which we read in the field. We centrifuged blood preserved in PPTs at 3000***g*** for 15 min in the field and aliquoted plasma into sterile 1.2 ml cryogenic vials containing 2× DNA/RNA Shield (Zymo Research, Irvine, CA, USA) at a 1 : 1 ratio to preserve nucleic acids and inactivate viruses [51,52]. We stored samples at −20 °C, shipped them on ice to the USA and kept them at −20 °C until further analysis.

### Viral nucleic acid extraction and sequencing

We performed total nucleic acid extraction using laboratory protocols validated for the detection of blood-borne viruses of mammals [40,53, 54]. Briefly, we first centrifuged 500 μl of plasma + buffer at 10 000***g*** for 10 min to remove debris. We then extracted nucleic acids from the remaining supernatant using the QIAamp MinElute Virus Spin Kit (Qiagen, Hilden, Germany) following the manufacturer’s instructions, but without carrier RNA or nucleases. We synthesized first-strand cDNA using the SuperScript IV Synthesis Kit (Thermo Fisher Scientific, Waltham, MA, USA) with random hexamers and second-strand cDNA using the NEBNext Ultra II Non-Directional RNA Second Strand Synthesis Module (New England Biolabs, Ipswich, MA, USA). We prepared DNA libraries with the Nextera XT DNA Library Preparation Kit (Illumina, San Diego, CA, USA) and sequenced them on the Illumina MiSeq platform (MiSeq Reagent Kit v3 300×300 read length, paired end). Blank samples (reagents only) were used as negative controls.

### Bioinformatics and virus identification

We trimmed reads of low quality (the Phred quality score <30) and short length (<50 bp) using CLC Genomics Workbench v. 23.0.2 (Qiagen). We then conducted *in silico* removal of reads matching known contaminants, ribosomal sequences and the assembled domestic dog reference genome (GenBank GCF_011100685.1; [55]). We assembled the remaining reads from each sample into contiguous sequences (contigs) using metaSPAdes v.3.15.5 [56]. We clustered contigs from each individual using CD-HIT-EST [57] to find the longest representative contig with ≥90 % nucleotide (nt) identity. We retained all representative contigs ≥500 nt and queried them using a six-frame translation against the NCBI non-redundant protein database using DIAMOND [58]. We examined all matches to virus sequences with *E*-values <10^−10^ manually by conducting blastn and blastx searches [59] to the full GenBank nucleotide and protein databases, respectively, and by examining the arrangement of open reading frames of putative viruses using an ORF finder [60]. We excluded bacteriophage sequences from further analysis, as our goal was to examine viruses capable of infecting vertebrates.

### Phylogenetics and viral sequence comparisons

To infer phylogenetic relationships among viruses, we first generated multiple alignments of partial ORF1 (Pfam accession PF02956) amino acid sequences (*Anelloviridae*), NS1 (Pfam accession PF01057) amino acid sequences (*Parvoviridae*) and VP3(T2) (Pfam accession PF01700) amino acid sequences (*Sedoreoviridae*) from the viruses identified in this study (see below) and known, non-identical viruses within the clade to which each virus belongs based on initial GenBank queries, using the muscle algorithm [61] implemented in NGPhylogeny [62]. We then edited the resulting alignments to remove poorly aligned regions using trimAl [63]. We inferred maximum-likelihood phylogenetic trees using PhyML with Smart Model Selection and 1000 bootstrap replicates [64,65]. We displayed the resulting phylogenetic trees in FigTree v. 1.4.4 [66].

### Statistical analyses

We calculated prevalence as the proportion of dogs in which a viral nucleotide sequence was detected. We calculated richness as the number of viruses present in an individual dog. We calculated abundance as log_10_ viral reads per million per kilobase of target sequence (log_10_vRPM/kb), which is a normalized, validated measure correlated with quantitative real-time PCR (qRT-PCR) data [67]. We conducted statistical analyses to examine differences among populations with respect to these measures using Kruskal–Wallis tests to account for the non-Gaussian distributions of variables. We similarly examined associations between viral richness and abundance and characteristics of the dogs (wildlife contact, physical examination parameters and blood evaluation data; see above) using Kruskal−Wallis tests, chi-square tests, Fisher’s exact tests, Mann–Whitney *U* tests and Spearman rank-order correlation analyses. We performed statistical analyses in Prism 10.2.0 (GraphPad Software, Boston, MA, USA).

## Results

### Characteristics of the three dog populations

Characteristics of dogs in the three populations followed expectations based on the study design (Table 1). The 48 dogs included in the study ranged in age from 0.4 to 13 years, and the average age did not differ significantly among populations [Kruskal−Wallis test statistic=4.441 (3, 42); two-tailed *P*=0.1086; Table 1]. Owners reported that 37.5 % of dogs in Bundibugyo District had ever been vaccinated against any diseases (including rabies) compared to 25.0 % in Kabarole District and 100 % in Wakiso District (chi-square 13.79, 2 degrees of freedom; two-tailed *P*=0.0010). Owners reported that 4.2 % of dogs in Bundibugyo District were routinely de-wormed (typically with ivermectin) compared to 8.3 % in Kabarole District and 100 % in Wakiso District (chi-square 35.29, 2 degrees of freedom; two-tailed *P*<0.0001). Owners reported that a much higher proportion of dogs in Bundibugyo District hunted wildlife (79.2 %) than did those in Kabarole (8.3 %) or Wakiso (0 %) districts (chi-square 26.06, 2 degrees of freedom; two-tailed *P*<0.0001). Owners also reported that a higher proportion of dogs in Bundibugyo District were fed wildlife (83.3 %) than in Kabarole (41.7 %) or Wakiso (0 %) districts (chi-square 22.96, 2 degrees of freedom; two-tailed *P*<0.0001). Among dogs that were fed wildlife, those in Bundibugyo District were fed wildlife approximately 26 times more often (90.95 days per year) than those in Kabarole District (3.50 days per year) (Mann–Whitney *U*=0; two-tailed *P*<0.0001).

**Table 1.** Characteristics of three populations of dogs in Uganda

Parameter*	Wakiso District (*n*=12)	Kabarole District (*n*=12)	Bundibugyo District (*n*=24)
Age (years)	4.8±1.0^a^	4.6±1.2^a^	2.7±0.7^a^
Hunt wildlife	0^a^	8.3 (0, 26.7)^b^	79.2 (61.7, 96.7)^c^
Fed wildlife	0^a^	41.7 (9.0, 67.3)^b^	83.3 (73.4, 99.4)^c^
Fed wildlife (days per year)	0^a^	3.5±2.2^b^	90.95±18.1^c^
Body condition score	3.4±0.7^a^	2.4±0.9^b^	2.5±0.7^b^
Body temperature (°C)	38.4±0.4^a^	37.0±0.6^b^	36.8±0.6^b^
Packed cell volume (%)	48.5±1.9^a^	38.0±2.1^b^	35.7±1.3^b^
Total protein (g/dl)	7.3±0.2^a^	8.0±0.3^a^	7.6±0.3^a^
White blood cell count (cells/µl)	9591±879^a^	13 564±1119^b^	14 532±659^b^

*Values are either mean±standard error or per cent (95 % confidence interval). Different superscript letters indicate statistically significantly different values for each parameter.

The BCS among dogs ranged from 1 to 4 and was highest for dogs in Wakiso (3.42±0.67 SD) followed by Bundibugyo (2.52±0.67 SD) and Kabarole (2.42±0.90 SD) districts [Kruskal–Wallis test statistic=11.59 (3, 47); two-tailed *P*=0.0030; Table 1 and Fig. S1, available in the online Supplementary Material]. Similarly, body temperature (degrees Celsius) was significantly higher for dogs in Wakiso (38.4±0.41 SD) than for dogs in Kabarole (37.0±0.60 SD) or Bundibugyo (36.8±0.65 SD) districts [Kruskal–Wallis test statistic=19.34 (3, 43); two-tailed *P*<0.0001; Table 1 and Fig. S1]. PCV (per cent) was also higher for dogs in Wakiso (48.5±6.22 SD) than for dogs in Kabarole (38.0±7.35 SD) or Bundibugyo (35.7±5.57 SD) districts [Kruskal–Wallis test statistic=15.71 (3, 42); two-tailed *P*=0.0004; Table 1 and Fig. S1]. In fact, 9 (75.0 %) dogs in Bundibugyo District and 2 (16.7 %) dogs in Kabarole District had PCV less than 35 %, indicating clinical anaemia [68], compared to zero clinically anaemic dogs in Wakiso District. TP (grams per decilitre) did not differ significantly among dogs in Wakiso (7.35±0.79 SD), Kabarole (8.02±1.06 SD) or Bundibugyo (7.64±1.23 SD) districts [Kruskal–Wallis test statistic=3.09 (3, 41); two-tailed *P*=0.2132; Table 1 and Fig. S1]. WBC (cells per microlitre) were markedly lower for dogs in Wakiso (9591±2916 SD) than for dogs in Kabarole (13 564±3710 SD) or Bundibugyo (14 532±2872 SD) districts [Kruskal–Wallis test statistic=13.03 (3, 41); two-tailed *P*=0.0015; Table 1 and Fig. S1].

### Viruses identified

After quality and length trimming, we obtained a total of 92 305 823 sequences (average 1 883 792 sequences per dog) from which we identified contigs representing 46 viruses in three families (*Sedoreoviridae*, *Parvoviridae* and *Anelloviridae*) based on their closest matches in GenBank (Table S1). Of these, 2 represented known viruses and 44 represented putatively novel viruses, as evidenced by low similarity to other viruses in GenBank or blast matches over only short regions (Table 1). We named the 44 novel viruses using the identifier ‘ucalufa’ to indicate their origin in Uganda in domestic dogs (*Canis lupus familiaris*). None of these viruses appeared in negative controls.

**Fig. 2.**
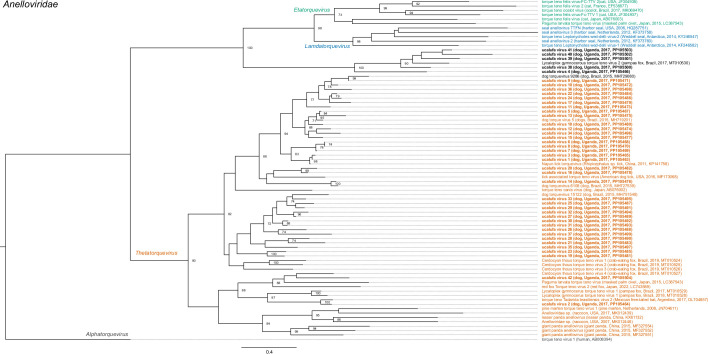
Maximum-likelihood phylogenetic tree of anelloviruses based on an alignment (455 positions) of partial ORF1 amino acid sequences. Taxa are colour coded to indicate clades corresponding to the recognized genera. The tree is outgroup rooted. Taxon names are followed by host, country, year and GenBank accession number in parentheses, where available. The taxon names of viruses identified in this study are in bold. Numbers beside nodes indicate bootstrap values (per cent; only values ≥50 % are shown); the scale bar indicates nucleotide substitutions per site.

**Fig. 3.**
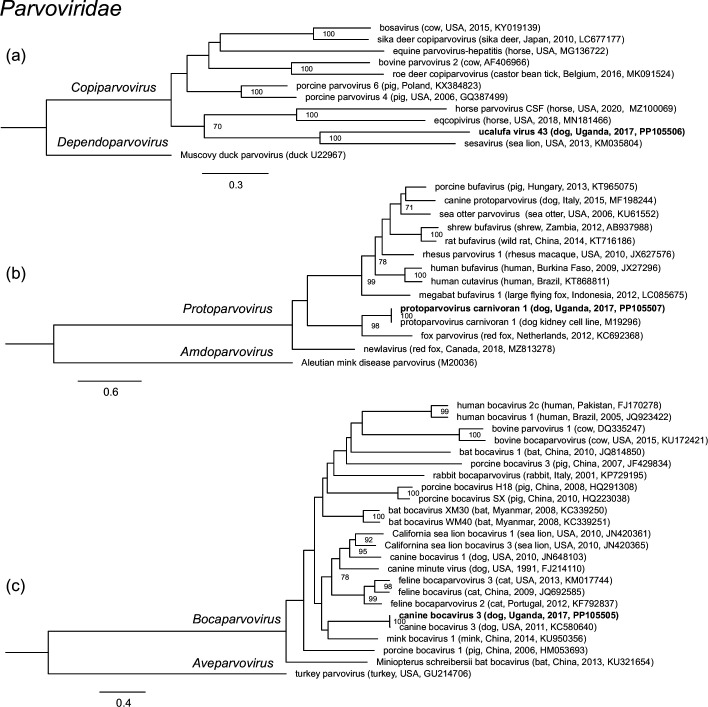
Maximum-likelihood phylogenetic trees of parvoviruses based on alignments ((a) 636 positions, (b) 406 positions and (c) 593 positions) of partial NS1 amino acid sequences. Clades corresponding to recognized genera are indicated. Each tree is outgroup rooted. Taxon names are followed by host, country, year and GenBank accession number in parentheses, where available. The taxon names of viruses identified in this study are in bold. Numbers beside nodes indicate bootstrap values (per cent; only values ≥50 % are shown); scale bars indicate nucleotide substitutions per site.

**Fig. 4.**
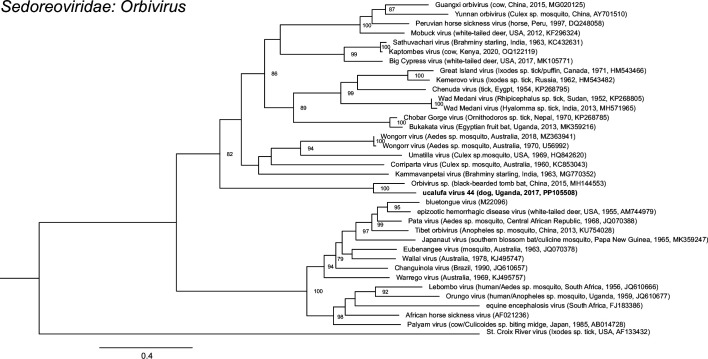
Maximum-likelihood phylogenetic tree of orbiviruses based an alignment (815 positions) of partial VP3 (T2) amino acid sequences. The tree is outgroup rooted. Taxon names are followed by host, country, year and GenBank accession number in parentheses, where available. The taxon name of the virus identified in this study is in bold. Numbers beside nodes indicate bootstrap values (per cent; only values ≥50 % are shown); the scale bar indicates nucleotide substitutions per site.

All viruses could be provisionally classified into family (Table S1). The majority (42/46; 91.3 %) were members of the family *Anelloviridae*, of which 37 were members of the genus *Thetatorquevirus*, whereas 5 could not be classified into genus (Table S1 and Fig. 2). Three viruses belonged to the family *Parvoviridae* and were most closely related to members of the genera *Protoparvovirus*, *Bocaparvovirus* and *Copiparvovirus* (Table S1 and Fig. 3). The first of these was identified as canine bocavirus 3 (CnBoV3) by virtue of its being 99.13 % identical to the reference genome across the length of the contig. The second, ucalufa virus 43, was distantly related (69.5 % nucleotide identity) to sesavirus, a member of the genus *Copiparvovirus* detected in a California sea lion (*Zalophus californianus*) in the USA in 2013 [69]. The third was identified as protoparvovirus carnivoran 1 (CPV1), commonly known as ‘canine parvovirus’, because it is 100 % identical to known variants of CPV1. Finally, ucalufa virus 44 was identified as a putatively novel orbivirus (*Sedoreoviridae: Orbivirus*) sharing 70.1 % identity across a nearly-coding-complete nucleotide segment sequence with a virus detected in a black-bearded tomb bat (*Taphozous melanopogon*) in China in 2015 (Table S1 and Fig. 4).

Phylogenetic analyses revealed the anelloviruses identified in Ugandan dogs to be widely dispersed within the clade representing the genus *Thetatorquevirus* (Fig. 2). These viruses tended to form subclades of similar sequences, each most closely related to previously identified viruses of domestic dogs or wild canids or to viruses in tick vectors in Asia, North America and South America (Fig. 2). The exception was ucalufa virus 2, which was sister taxon to a virus found in a Mexican free-tailed bat (*Tadarida brasiliensis*) in Argentina in 2017 [70]. Anelloviruses not in the *Thetatorquevirus* clade were monophyletic and clustered with a virus identified in a Pampas fox in Brazil in 2017, in a clade that has not yet been classified to genus (Fig. 2). Phylogenetic analyses of the three parvoviruses identified in Ugandan dogs revealed them each to belong to subclades containing other parvoviruses of carnivores, with the novel virus ucalufa virus 43 being most closely related to sesavirus from a sea lion but not particularly closely related to any other known virus in the genus *Copiparvovirus* (Fig. 3). Phylogenetic analysis of ucalufa virus 44 revealed it to be the sister taxon to the aforementioned orbivirus from a black-bearded tomb bat within a subclade of viruses from various other bats, hoofed mammals and insect vectors (Fig. 4). Of note, no other viruses within the genus *Sedoreoviridae*, which contains African horse sickness virus and bluetongue virus, are typically associated with carnivore hosts.

### Viral prevalence

**Fig. 5.**
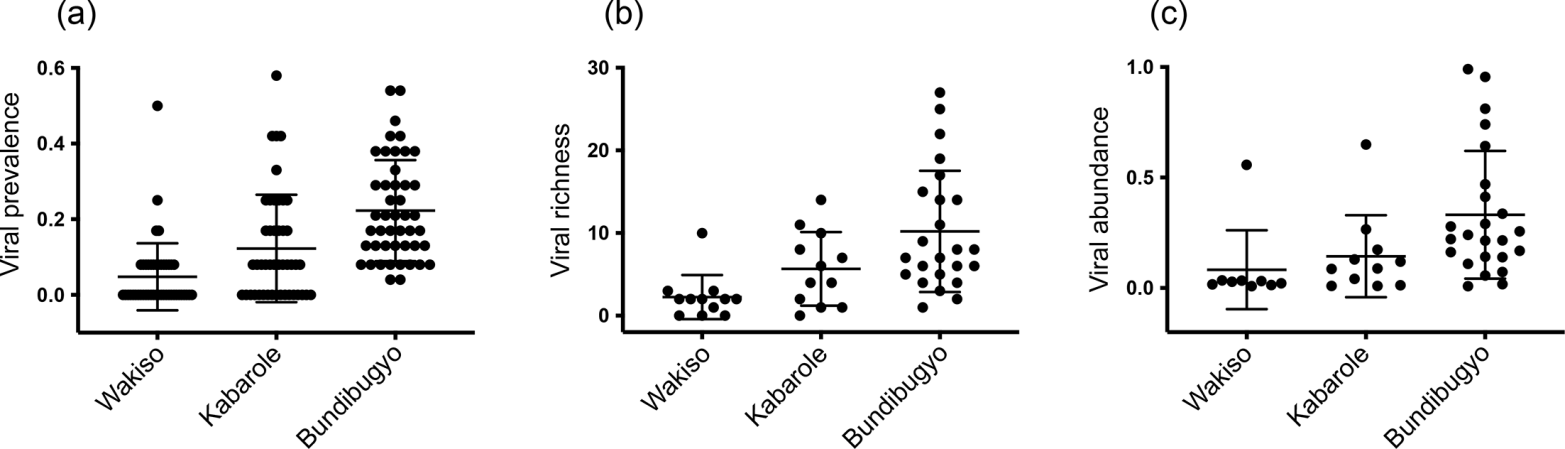
Viral prevalence (proportion of dogs infected; 46 viruses), richness (number of viruses per dog; 48 dogs) and abundance (log_10_vRPM/kb; 44 dogs in which at least one virus was detected) in three populations of dogs in Uganda. The three populations were selected to span a gradient of contact with wildlife, from Wakiso (low contact; *n*=12) to Kabarole (medium contact; *n*=12) to Bundibugyo (high contact; *n*=24). Lines and error bars are means and standard deviations, respectively.

All 46 viruses (100 %) were identified in dogs from Bundibugyo District, compared to 29 viruses (63.0 %) in Kabarole District and 18 viruses (39.1 %) in Wakiso District (chi-square 12.84, 2 degrees of freedom; two-tailed *P*=0.0016) (Table S1). Notably, 13 viruses (canine bocavirus 3, protoparvovirus carnivoran 1 and ucalufa viruses 5, 6, 12, 15, 18, 20, 24, 27, 32, 37 and 43) were identified only in Bundibugyo District. Prevalence of each virus among all dogs in the study ranged from 2.1 to 92.0 % (Table S1). The average prevalence of viruses within each population was highest in Bundibugyo District (22.3±13.4 % SD), followed by Kabarole (12.3±14.2 % SD) and then by Wakiso (4.8±0.9 % SD) districts [Kruskal–Wallis test statistic=47.26 (3, 138); two-tailed *P*<0.0001] (Fig. 5a).

### Viral richness

Viral richness ranged from 0 to 27 viruses per dog and was markedly higher in Bundibugyo District (10.21±7.33 SD) than in Kabarole (5.67±4.46 SD) or Wakiso (2.25±2.67 SD) districts [Kruskal–Wallis test statistic=15.88 (3, 48); two-tailed *P*=0.0004] (Fig. 5b). Six dogs from Bundibugyo District had higher viral richness than dogs from elsewhere (richness values of 27, 25, 22, 19, 17 and 15, shown as outliers in Fig. 5b). This trend of highest viral richness in dogs from Bundibugyo District, followed by Kabarole and then by Wakiso districts, was driven by the following 10 viruses, each of which was significantly more prevalent in Bundibugyo District than in the other districts (Fisher’s exact *P*<0.05 in all cases; Table S1): the anelloviruses ucalufa virus 9, 10, 11, 13, 18, 23, 27, 30 and 31 and the novel orbivirus ucalufa virus 44 (Table S1).

Viral richness was strongly positively correlated with the number of times per year that a dog was fed wildlife (Spearman *r*=0.4740; two-tailed *P*=0.0007). Viral richness was negatively correlated with BCS (Spearman *r*=−0.3131; two-tailed *P*=0.0321), with body temperature (Spearman *r*=−0.3706; two-tailed *P*=0.0144) and marginally with PCV (Spearman *r*=−0.3173; two-tailed *P*=0.0406).

### Viral abundance

Viral abundance ranged 123.9-fold, from 0.008 to 0.991 log_10_vRPM/kb. Viral abundance (only infected dogs were included in the analysis) was higher in dogs from Bundibugyo District (0.33±0.29 SD) than in dogs from Kabarole (0.14±0.19 SD) or Wakiso (0.08±0.18 SD) districts [Kruskal–Wallis test statistic=12.55 (3, 44); two-tailed *P*=0.0019] (Fig. 5c). Four dogs from Bundibugyo had higher viral abundance than dogs from elsewhere (viral abundance values of 0.955, 0.991, 0.812 and 0.741, shown as outliers in Fig. 5c).

Viral abundance was strongly positively correlated with the number of times per year that a dog was fed wildlife (Spearman *r*=0.3763; two-tailed *P*=0.0018). Viral abundance was negatively correlated with PCV (Spearman *r*=−0.3743; two-tailed *P*=0.0206) and positively correlated with WBC (Spearman *r*=0.3733; two-tailed *P*=0.0229).

## Discussion

We studied domestic dogs in three populations in Uganda to test the hypothesis that dog viromes vary across a gradient of contact with wildlife. Demographic and behavioural data confirmed expectations from our study design. Seventy-nine per cent of dogs in Bundibugyo District were used for hunting of wildlife compared to 8% of dogs in the agricultural communities of Kabarole District and 0 % in dogs from the urbanized Wakiso District. Dogs in Bundibugyo District were fed wildlife 26 times more often per year than dogs in Kabarole District, and dogs in the Wakiso comparison population were never fed wildlife. The principal ethnic groups in Bundibugyo District who were owners of the dogs in our study were the Bwamba and Bakonjo. These groups practice traditional hunting of wildlife, including with dogs, and encounters between dogs and wildlife during hunting excursions are typically violent and bloody [26,45].

Dogs in Bundibugyo and Kabarole districts, which were free-roaming, were of generally poor health compared to dogs from Wakiso District, which were owned as pets and were not free-roaming. Rates of vaccination and de-worming were low in Bundibugyo and Kabarole districts (below 38 % and below 9 %, respectively), whereas rates of vaccination and de-worming were 100 % in Wakiso District. These differences reflect a lack of financial resources and veterinary care in the former two districts. It is therefore not surprising that BCS was low in Bundibugyo and Kabarole districts but higher and clinically normal in Wakiso District. Interestingly, body temperature was also lower in dogs from Bundibugyo and Kabarole districts than in dogs from Wakiso District. This finding may reflect nutritional status, in that malnourished dogs may lack adequate caloric intake for thermoregulation, especially when confronted with the need for upregulated immunological responses [71,72]. Our finding that PCV was lower in dogs from Bundibugyo and Kabarole districts than in dogs from Wakiso District may reflect a similar effect of nutrition, with iron deficiency leading to clinical anaemia in 75 and 17 % of dogs from these districts, respectively, compared to 0 % in Wakiso District [73]. Alternatively, low PCV may reflect anaemia of inflammatory disease [74], perhaps caused by the lack of regular anthelminthic treatment and consequent infection with canine hookworm (*Ancylostoma* spp. and *Uncinaria* spp.), which is endemic in the region [75], or by protozoan parasites of blood cells, such as reported for hunting dogs in Tobago [76]. Our observation that WBCs were higher in dogs from Bundibugyo and Kabarole districts than in dogs from Wakiso District may reflect a higher burden of infectious disease or higher rates of generalized inflammation (e.g. from physical injury) in dogs from the former two populations, as might be expected from the general risks of a free-roaming lifestyle.

Viral prevalence, richness and abundance are all co-varied with demographic and behavioural factors in ways that support our central hypothesis. All viruses were present in dogs from Bundibugyo District, whereas only 63 and 39 % of viruses were present in Kabarole and Wakiso districts, respectively. Average viral prevalence was approximately twice as high in Bundibugyo District than in Kabarole District and approximately four times as high as in Wakiso District. Both viral richness and abundance were markedly higher in dogs from Bundibugyo District than in the other two districts, with dogs from Kabarole District having intermediate viral richness and abundance and dogs from Wakiso District having low viral richness and abundance. This pattern parallels the gradient of wildlife contact among populations upon which our study was designed. These patterns may reflect contact with wildlife and viral transmission. However, many other factors also differ among the three dog populations, including ecology, ownership practices, access to veterinary care, nutrition and socioeconomics. Any of these could have affected viral infection by altering the probabilities of transmission or by altering the ability of dogs to resist or control infection. It is intriguing, however, that both viral richness and abundance were strongly positively correlated with the number of times per year that a dog was fed wildlife. Dogs fed wildlife in our study populations were typically fed the uncooked remnants of wild animals butchered for human consumption (often entire carcasses), offering a plausible mechanism for viral transmission.

Viral richness and abundance also co-varied with clinical health metrics in ways indicating that the health status of the dog modified viral infection. Specifically, dogs with lower BCS had higher viral richness, perhaps reflecting immunological compromise due to malnutrition [77]. This same mechanism might explain the paradoxical negative association between body temperature and viral richness (opposite in direction from what would be predicted based on a febrile response) – namely, that energy deficits can reduce both thermoregulatory ability and immune function [71,72]. A mechanism behind the negative association between viral abundance and PCV is not immediately apparent. However, viral abundance was positively correlated with WBC, as would be expected if dogs were mounting an immune response. Unfortunately, we were unable to perform differential blood cell counts in the field, which might have indicated whether WBCs were elevated in ways indicative of an antiviral immune response [78].

Dog serum viromes in our study were similar to those reported by Weber *et al.* [79], who applied metagenomics and real-time PCR to blood serum from 520 dogs from Brazil and identified parvoviruses (including a bocaparvovirus, a protoparvovirus and a relative of sesavirus) and anelloviruses in the genus *Thetatorquevirus* as the predominant sequence variants. Other studies focused on faecal and respiratory samples [80,83] have identified parvoviruses as common constituents of dog viromes but have not detected anelloviruses, which are blood-borne. Because anelloviruses cause no known clinical disease and are not well studied, the modes of transmission of these viruses remain incompletely understood [84,85]. The anelloviruses identified in our study were highly diverse but tended to cluster phylogenetically with anelloviruses from dogs and wild carnivores. This finding would be consistent with transmission from wild carnivores, which are hunted in our study populations. However, anelloviruses also cause persistent infections and are considered biomarkers of immunocompromise, in that they are known to ‘spike’ in immunocompromised people (e.g. organ transplant patients) [84,87]. It is therefore also plausible that the anelloviruses we identified naturally infect dogs and that the trends we observed reflect variation among dogs in their ability to control persistent viral infection. The latter explanation is likely for two of the three parvoviruses we identified, CnBoV3 and CPV1, which are known viruses of dogs. However, ucalufa virus 43 in the genus *Copiparvovirus*, distantly related to sesavirus from a sea lion, has no known close relatives and could have originated in a non-canine species. Distinguishing whether these patterns of anellovirus and parvovirus infection originate from increased transmission or reduced immunity would require additional research into the natural history of these agents.

A noteworthy finding of our study is the novel orbivirus ucalufa virus 44, with no known relatives that typically infect dogs or other carnivores. This virus was present in 25.0 % of dogs from Bundibugyo and 8.3 % of dogs from Kabarole but was absent from dogs in Wakiso. Among this virus’s congenerics, most is known about African horse sickness virus and bluetongue virus. African horse sickness virus is transmitted to equids by culicoid midges and can cause severe or even lethal febrile, cardiac and pulmonary diseases [88,90]. African horse sickness virus can also infect dogs, notably via the oral route through ingestion of infected horse meat [91,94], and it appears to infect wild African carnivores widely [95]. Bluetongue virus is also typically transmitted by culicoid midges to diverse vertebrates (primarily ruminants) around the world [92,96]. Like African horse sickness virus, it can also infect dogs and cause clinical disease through vector-borne transmission, possibly through ingestion of infected meat, or through contaminated vaccines [97,102], and it also appears to infect wild carnivores widely [103]. Importantly, several other lesser-studied orbiviruses are zoonotic (e.g. Corriparta virus, Changuinola virus, Kemerovo virus and Orungo virus) or can infect dogs [104]. It is therefore plausible that ucalufa virus 44 infected dogs in our study from another host, perhaps through direct contact, ingestion of infected meat or vector-borne transmission. Our observations that ucalufa virus 44 was only detected in dogs with wildlife contact and that viral richness and abundance increased with the number of times per year that a dog was fed wildlife lend credence to the idea that dogs may have acquired this virus by consuming its natural wildlife host. Identifying the natural host(s) of ucalufa virus 44 should be a research priority, and it would be prudent to include this virus in zoonoses surveillance efforts of people in the region, especially if they own hunting dogs.

Overall, our results support the hypothesis that dogs that frequently hunt wildlife are at increased risk for viral infection. The mechanism for this association could be increased transmission of viruses from wildlife due to contact between dogs and wildlife during hunting excursions or through ingestion of wildlife meat. Alternatively, the mechanism for this association could be a general impairment of immunity in dogs living in locations where wildlife hunting is common and where ecological and social conditions contribute to ill health and reduced immunity. In either scenario, our results indicate that risks of viral infection are elevated in dogs in locations where they contact wildlife frequently and where canine health is poor. This observation, in turn, is consistent with the idea that free-roaming dogs may serve as intermediary hosts for zoonoses, especially in ‘high-risk’ locations for zoonoses. In such locations, people may also be in poor health due to such factors as poor nutrition, a high burden of infection and immunocompromise (e.g. due to HIV) and limited access to health care. Such conditions are unfortunately common worldwide in locations with large and uncontrolled populations of free-roaming dogs. Monitoring people and their dogs in such areas for novel viruses might be an efficient strategy for early detection of spillover.

## Conclusion

Our findings support the hypothesis that free-roaming dogs in areas of high wildlife contact are at an increased risk of viral infection. Data on canine health show that this risk is modified by the health status of the dog. Other factors related to geography, ecology and socioeconomics could also explain the trends we have documented, and these are not mutually exclusive. Future studies of the natural history of the viruses we identified would be informative, as would quantifying how the nutritional, physiological and immunological condition of dogs might alter their ability to become infected or to control infection. We draw attention to a novel orbivirus detected only in populations of dogs with wildlife contact. Certain orbiviruses can infect dogs through ingestion of infected meat, and other orbiviruses are zoonotic. In aggregate, our findings support the notion that simultaneous monitoring of dogs and people in high wildlife contact areas might be an efficient strategy for early detection of spillover.

## supplementary material

10.1099/jgv.0.002011 Uncited Fig. S1.

10.1099/jgv.0.002011 Uncited Table S1.

## Abbreviations

BCS: body condition score
cDNA: complementary DNA
CnBoV3: canine bocavirus 3
CPV1: protoparvovirus carnivoran 1
DNA: deoxyribonucleic acid
EDTA: ethylenediaminetetraacetic acid
NCBI: National Center for Biotechnology Information
PCV: packed cell volume
PPT: plasma preparation tube
qRT-PCR: quantitative real-time PCR
RNA: ribonucleic acid
SARS-CoV-2: severe acute respiratory syndrome coronavirus 2
TP: total protein
WBC: white blood cell count
