# Supplementary figures and images for: Viruses of free-roaming and hunting dogs in Uganda show elevated prevalence, richness and abundance across a gradient of contact with wildlife
Source: J Gen Virol. 2024 Jul 24;105(7):002011. doi: 10.1099/jgv.0.002011 (PMC11316573; doi:10.1099/jgv.0.002011)

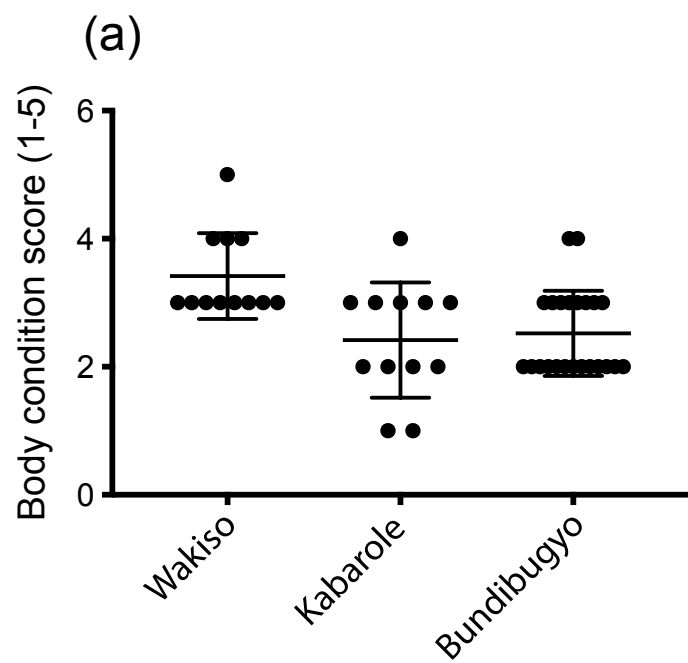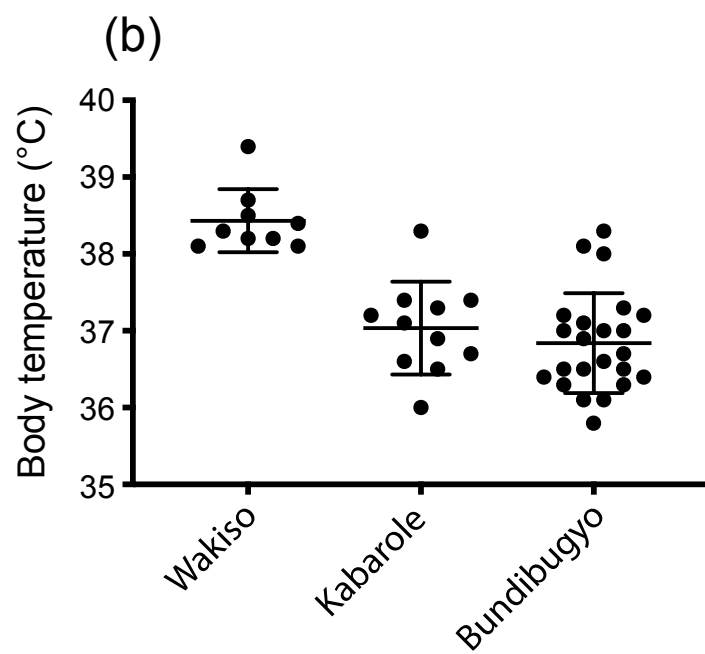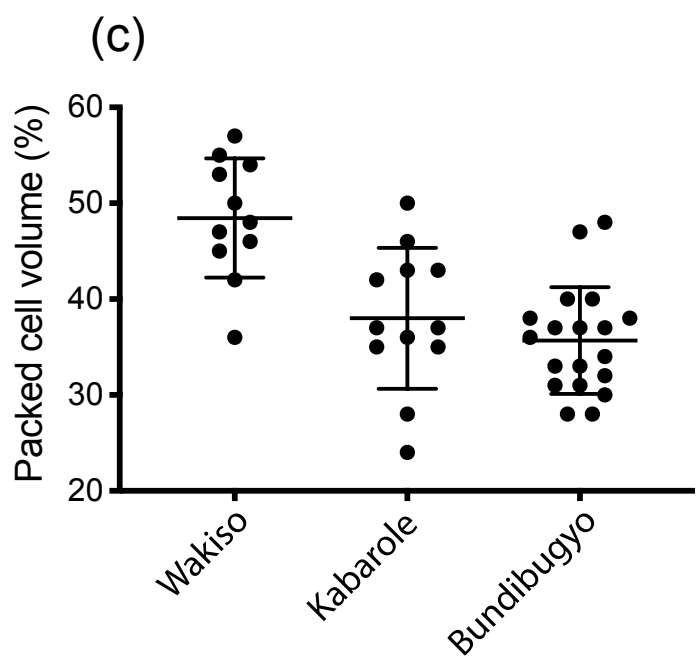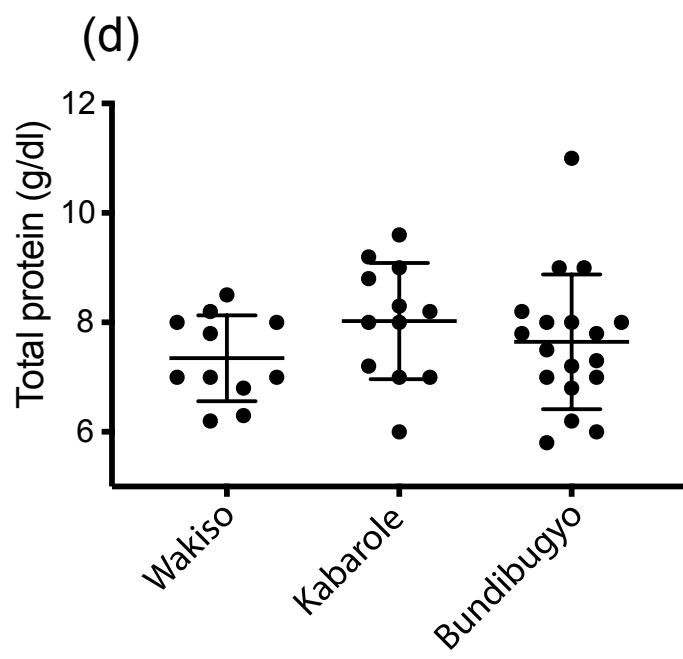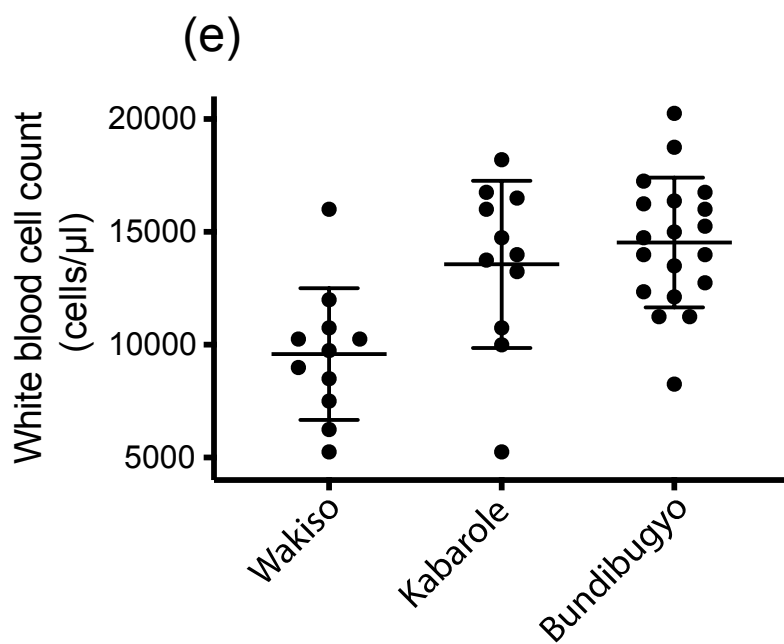

Supplement: Uncited Fig. S1. [file jgv-105-02011-s001.pdf]
